# Introduction of the Second Dose of Measles Containing Vaccine in the Childhood Vaccination Programs Within the WHO Africa Region – Lessons Learnt

**Published:** 2018-07-28

**Authors:** Balcha G Masresha, Richard Luce, Joseph Okeibunor, Messeret Eshetu Shibeshi, Raoul Kamadjeu, Amadou Fall

**Affiliations:** 1 WHO Regional Office for Africa. Brazzaville, Congo; 2 WHO Inter-country Support Team for Central Africa. Libreville, Gabon; 3 WHO Inter-country Support Team for East and Southern Africa. Harare, Zimbabwe; 4 UNICEF regional office for Eastern and Southern Africa. Nairobi, Kenya; 5 WHO Inter-country Support Team for Western Africa. Ouagadougou, Burkina Faso

**Keywords:** Measles-Containing-Vaccines, Measles, Second Dose, Post-Introduction Evaluation, African Region

## Abstract

**Background:**

WHO recommends all countries to include a second routine dose of measles containing vaccine (MCV2) in their national routine vaccination schedules regardless of the level of coverage with the first routine dose of measles containing vaccine (MCV1). As of Dec 2016, 26 countries in the African Region have introduced MCV2.

**Methods:**

We reviewed the WHO UNICEF coverage estimates for MCV1 and MCV2 in these countries, and the reports of the post introduction evaluation of MCV2 from 11 countries.

**Results:**

Twenty three countries have WHO/UNICEF estimates of MCV2 coverage available in 2015. Of these, 2 countries have coverage of ≥ 95% for both MCV1 and MCV2 while 5 countries have coverage of > 80% for both doses. Dropout rates of >20% MCV1 – MCV2 exist in 12 countries. Post-MCV2 introduction evaluations done in 11 countries from 2012 to 2015 showed that inadequate health worker training, insufficient sensitization and awareness generation among parents and suboptimal dose recording practices were common programmatic weaknesses that contributed to the low MCV2 coverage in these countries.

**Conclusion:**

MCV2 coverage remains low as reflected in large drop-out rates in most countries. Higher MCV2 coverage is necessary to sustainably achieve the regional measles elimination goal. National immunization programs must improve implementation of MCV2 using the standard introduction and evaluation guidelines available for EPI program planning.

## Introduction

In 2011, the African Region of the World Health Organization (WHO) adopted a Regional measles elimination goal for 2020, and is implementing the recommended strategies, to attain the following targets: (a) measles incidence of less than 1 case per million population at national level; (b) at least 95% measles immunization coverage at national level and in all districts; (c) at least 95% coverage in all scheduled measles SIAs, and in outbreak response immunization activities; (d) at least 80% of districts investigating one or more suspected measles cases within a year, and a non-measles febrile rash illness rate of at least 2 per 100,000 population at national level^1^, ^2^.

Through the implementation of the recommended strategies, countries in the Region have experienced 85% reduction in measles mortality, from an estimated 414,500 deaths in 2000 to 61,600 in 2015^3^.

In 2009, WHO recommended countries to introduce the second dose of measles vaccine in the routine immunization schedule (MCV2) once they have achieved ≥80% coverage of MCV1 at the national level for 3 consecutive years as determined by the most accurate data available^4^. However, MCV2 introduction policy was revised in April 2017, recommending that countries to include MCV2 in their national vaccination schedules regardless of the level of MCV1 coverage^5^. WHO operational guidelines are available to assist countries comprehensively prepare the introduction of news vaccine including MCV2 into routine vaccination programs^6^, ^7^.

WHO guidelines further recommend that countries should conduct a post-introduction evaluation (PIE) exercise, within 6 to 12 months after the introduction of any new vaccine, to assess the process of introduction and correct deficiencies. The evaluation can be conducted separately are as part of a comprehensive Expanded Program on Immunisation (EPI) program review. The PIE is led by the Ministry of Health, and is supported by a team of experts from partner agencies. The methodology of the PIE typically includes a desk review of plans, guidelines, tools, data, as well as field visits including interviews of immunization program managers, cold chain officers, and health workers at different administrative levels^8^. The PIE provides a very good opportunity to build upon program strengths, and to rapidly identify programmatic gaps that should be addressed in order to assure a smooth roll out and high coverage.

We analyzed MCV2 coverage in 23 countries with available coverage estimates, and summarized the findings of post-introduction evaluation exercises done in eleven countries.

## Methods

National MCV1 and MCV2 coverage, as calculated by the national immunization program using administrative methods of dividing the total number of doses administered to children in the target age group, is reported annually to the WHO and UNICEF. WHO and UNICEF generate estimates of coverage for all antigens based on the reported data, as well as data from surveys and other sources of data validation. These WHO-UNICEF estimates of national coverage (WUENIC) for MCV1 and MCV2 coverage for 2015 were reviewed for countries that have introduced MCV2 into their routine immunization schedules as at the end of 2015^9^. Furthermore, we reviewed post-introduction evaluation reports from 11 countries that introduced MCV2 between 2012 and 2015^10^–^18^ and extracted key details of the various aspects of the preparation and implementation of MCV2 introduction including planning, training, development of guidelines and monitoring tools, demand generation, coverage monitoring, cold chain capacity, knowledge of health workers, parent/ caretaker awareness, as well as service delivery and supportive supervision in the months following vaccine introduction^8^.

## Results

By the end of 2015, MCV2 had been introduced in 26 countries in the WHO Africa Region. Of these, 23 countries reported coverage data to WHO and UNICEF and have coverage estimates available for 2015. Mozambique, Sierra Leone and Zimbabwe introduced MCV2 in late 2015 and did not report coverage since vaccination was implemented for less than full year.

Six countries had MCV2 in their childhood vaccination schedules as of 2011; an additional 20 countries introduced MCV2 in the period 2012-2015. The Regional MCV1 and MCV2 coverage levels for the past 10 years are given in Table 1. Regional MCV2 coverage remained less than 10% until 2014. Most countries provide MCV2 between 15 and 18 months of age; however, Algeria, Mauritius and Seychelles provide MCV2 at school entry and South Africa provides it at 12 months of age. In 2015, MCV2 coverage levels were 95% and above in Cape Verde, Seychelles and Algeria. MCV2 coverage was 80% - 90% in 5 countries and less than 60% in 8 countries (Table 2). The median drop-out rate between MCV1 and MCV2 was 21% in 2015. Five countries had drop-out rates of less than 10%, and 12 countries had drop-out rates in excess of 20%. (Table 2)

Between 2013 and 2016, post-introduction evaluations for MCV2 were conducted in 11 countries. (Table 3) The timing of the evaluations ranged from 9 to 37 months after MCV2 introduction. Some of these evaluations were part of comprehensive Expanded Program on Immunisation (EPI) reviews, while others targeted multiple newly introduced vaccines. The major findings from the MCV2 PIE activities in these 11 countries are outlined below.

### Burundi

The MCV2 post introduction evaluation was combined with the rotavirus PIE, and was conducted in 23 of the 45 districts in the country and included interviews with 144 mothers with children in the vaccination program target population.

MCV2 introduction benefitted from high level political commitment and programmatic support. A copy of the MCV2 introduction plan was available in 12/23 (52%) of districts and training materials present in 38/67 (57%) health centers. Training of health workers was conducted in 56/67 (83%) of health centers an average of 8 days prior to introduction. Approximately 50% of health centers had updated tally sheets, vaccination registers and child health cards to reflect MCV2.

The MCV2 introduction plan was available in 12/23 (52%) districts visited. The PIE documented that training of health workers on MCV2 was conducted in 84% prior to introduction. Training and reference materials on MCV2 were available in 38/67 (57%) of health facilities visited. Cold chain capacity was not reported as a constraint to MCV2 introduction. Vaccination with MCV2 was not offered every day in every health center, particularly in rural areas and 22/67 health centers (33%) did not have any MCV2 administrative coverage data for 2013 which was the year of introduction.

Data on vaccine wastage was available in 16 health facilities for 2012 and 2013 where wastage was reported as between 1% and 53%. Of these, 13 health facilities found that wastage had increased between 1% and 25% after introduction. As a result, the health centers reported reducing the number of vaccination days per week and waiting until 10 children were present before opening a vaccine vial. Only 2 minor cases of AEFI were reported in association with MCV2 introduction.

Radio, television and promotional videos were used to inform the population about MCV2 introduction. As a result, 80% of the interviewed parents reported knowing that children need 2 doses of MCV to be fully protected against measles.

A mean of 2.6 supervisory visits were conducted in the 6 months prior to introduction. Only 5/67 health facilities (7%) did not have a written report of the most recent supervisory visit available during the evaluation.

### Eritrea

In preparation for the MCV2 introduction, a review of vaccination monitoring and data collection tools was done with the involvement of child health, nutrition and other child survival programs. There was significant expansion of the cold chain at the national and regional levels as part of the preparations for the introduction of MCV2 and Rotavirus vaccines. The in-country vaccine distribution mechanism did not change with these new vaccines, and dry storage facilities had enough space to accommodate the new vaccines.

A series of training-of-trainers sessions were cascaded from the national to the lower administrative levels 2 weeks before the launch of MCV2. Health care workers in visited health facilities demonstrated adequate knowledge regarding MCV2.

In the health facilities visited, tally sheets, registers, ledgers, vaccination cards to document MCV2 were widely available. However, at national level the immunization field guide and national level summary sheet were not yet updated to include MCV2.

Social mobilization was done through radio, television and community groups preceding the introduction of MCV2 and Rotavirus vaccines. However, greater attention was focused on rotavirus vaccine introduction and the PIE documented a limited number of promotional materials on MCV2 since it was considered one of the traditional vaccines.

Approximately 84% of the health facilities visited reported receiving one or more supportive supervision and monitoring visits from the district and Zoba levels in the 6 months preceding the post-introduction evaluation.

### Gambia

In the Gambia, the decision to introduce MCV2 involved the review and endorsement of the introduction plan by the ICC. The 7 regions of the country did not have their own introduction plans but followed and implemented the national plan. Data recording systems and collection tools including tally sheets, immunization registers, infant welfare cards, were all updated to include MCV2. However, the national EPI policy document was not updated to include guidance on MCV2 use.

The training materials were adapted from the WHO guideline templates. Training was conducted for two days in each region. However, only 7 of the 23 health facilities reported having guidelines/ training materials. Health care workers were found to be knowledgeable about the justification, policy and technical issues around MCV2 introduction, but some of the health workers were not able to calculate coverage rates, drop-out and wastage rates.

The functional cold chain space was adequate to accommodate the addition of measles doses needed for the introduction of MCV2. Vaccine management and storage was found to be generally satisfactory. Storage volume and space for measles vaccine, needles & syringes and safety boxes were increased at all levels.

Community sensitization was conducted prior to and during introduction of the vaccine. But, 39 out of the 46 mothers interviewed during the evaluation were not aware of the availability and importance of MCV2 in the second year of life.

Supportive supervisory visits were conducted by the central and regional levels as 87% of health facilities visited during the PIE reported having one or more supervisory visits during the preceding six months. However, these visits were irregular and written feedback not given.

### Ghana

Prior to MCV2 introduction, Ghana completed a revision of the vaccine monitoring and data collection tools and training materials. Training was conducted in a cascaded manner starting with the central level followed by the district level using the WHO guidelines. Updated monitoring charts were available in health facilities. Nearly 60% of the health facilities visited had fact sheets on MCV2 and approximately 30% had updated child health record books. Most health facilities used multiple communication channels to inform parents about new vaccines. Health workers were able to respond question as necessary when they arose.

Regular supportive supervision was being conducted from the national and regional levels in the 6 months following MCV2 introduction. However, some health facilities still had outdated vaccine coverage monitoring tools. Health workers’ understanding of the parameters for the calculation of MCV2 coverage and drop-out rates was weak.

### Kenya

In Kenya, the Inter-Agency Coordinating Committee (ICC) endorsed MCV2 introduction and the national measles Technical Advisory Group provided technical input. The comprehensive multi-year costed immunisation plan document (cMYP) was updated to incorporate the investment requirements for the introduction of MCV2 and Rotavirus vaccines. Kenya used local funding resources to introduce MCV2.

MCV2 introduction guidelines were prepared by the national level. However, during the PIE, 12 of the 23 health facilities visited reported having received no guidelines, posters or teaching materials for MCV2 introduction. Kenya implemented on-job sensitization to train health workers. Training materials for MCV2 introduction were inadequate at the operational level in all counties.

Cold chain space was expanded in preparation for the introduction of new vaccines. However, at health facility level, the evaluation documented a limitation of cold chain space which required increased frequency of vaccine delivery to meet the vaccine demands, with cost implications for the county level.

For MCV2 introduction, IEC materials were developed at the national level, however no financial support was available to print and to distribute them. Demand generation activities to raise awareness among parents were limited. Direct inter-personal communication between health workers and parents was used as a primary means to create awareness about MCV2 during contacts in health facilities.

Almost all the health facilities and counties visited had up-to-date summary and tally sheets. But the available vaccination coverage monitoring charts did not include options for monitoring the coverage of MCV2 or rotavirus vaccine. Vaccine registers had not yet been updated to include MCV2 and RV.

The evaluation found that only 74% of the visited health facilities provided measles vaccine on daily basis, even though the national policy recommends daily vaccination service delivery. The majority of health workers interviewed did not know what figures to use as the denominator for MCV2 coverage monitoring.

MCV2 introduction did not take place nationwide at the same time. It was delayed until 2014 in two counties. The county and national levels had not reported MCV2 coverage officially in 2013 and 2014.

### Malawi

The Malawi cMYP was updated to include MCV2 and injectable polio vaccine (IPV) and the national EPI policy document includes MCV2. The cold chain capacity was assessed and considered adequate previous to the introduction of MCV2. There were no cold chain capacity limitations in the cold stores and health facilities visited.

MCV2 introduction did not occur at the same time across the country. The cascaded training took place prior to the introduction in the respective districts/health facilities.

All data collection and monitoring tools were updated and distributed to health facilities prior to the introduction. Most health facilities had the updated child health passport, and registers. Monitoring charts were available but were up-to-date in only 3/17 health facilities and were not used at all at the district level. Five out of 17 health facilities were using the correct denominator figures to monitor MCV2 coverage. Half of the interviewed health workers were able to calculate MCV2 coverage.

Various communication and mobilization strategies were implemented prior to the introduction of the MCV2 at all levels, with the engagement of professional societies and communities.

Health workers did not have a clear understanding of the policies regarding the vaccination of “sick” children; what to do if a child is older than the target age of 15-23 months; whether to open a vaccine vial if only one child is present; whether to administer missing “infant” antigens to children coming for MCV2 or how to document MCV2 in the new registers by age category. Very few planned supervisory visits were conducted from the district and national levels to the health facilities visited.

### São Tomé and Príncipe

The evaluation team visited 4 of 7 districts and 16 health centers. It included interviews with 72 mothers with children in the vaccination program target population. Fifteen vaccination service delivery sessions were also observed. The launch of MCV2 received high level attention from the government and was officially launched in a ceremony presided by the Health Minister.

The country updated its immunization policy document as well as data collection and documentation tools and the national immunization calendar prior to introduction. An introduction plan was available in all districts evaluated. Training was conducted in all districts and health centers an average of 8 days prior to introduction. The duration of training varied from 4 hours to 2 days. The number of vaccination sessions varied between once every 2 weeks to every day in the urban areas. This was reported as a common practice to reduce vaccine wastage. MCV2 vaccination status was verified by health workers in all vaccination sessions observed. The use of mobile vaccination teams in community was a strategy used as an outreach activity to ensure that routine doses of all vaccines were administered to children who had not returned to health centers to receive doses of the routine calendar.

Cold chain capacity was adequate to accommodate MCV2 doses. At the national level, MCV wastage was reported at 31.8% in 2013. At the district level, data were available from 2 districts and each experienced a reduction in wastage after MCV2 introduction; declining from 26.6% to 2013 to 13.2% in 2014 in one and from 31.8% to 29.8% in the second health facility. Reluctance to open vaccine vials to avoid vaccine wastage was reported by staff in 3/4 (75%) of districts and 8/16 (50%) of health centers. Monitoring of MCV1 and MCV2 using monthly coverage charts was verified in 2/4 (50%) of districts and in only 1/16 (6%) of health centers. Of mothers surveyed, 46/72 (74%) knew that children need two doses of MCV to be fully protected against measles; 26/62 (42%) obtained this information from a nurse. Vaccination cards were available for 61/62 (98%) of parents interviewed.

### Senegal

The plan for the introduction of the MR, Rotavirus, Injectable Polio Vaccine (IPV) was included in the cMYP 2014-2018. Senegal introduced MCV2 as MR2 in August 2014, about 15 months after the MR catch-up SIAs done in November 2013. A training guide and technical notes were developed and shared with the operational level, and cascade training completed at least a month before the introduction of the vaccines.

The data collection tools including vaccination registers, tally sheets, vaccination cards, stock registers, monthly reporting forms, were revised and distributed before the introduction of the vaccines. In addition, the routine vaccination database had been updated to accommodate the new vaccines.

Communication tools and messages were developed in the form of posters, leaflets, and memory aids. Intensive social mobilisation activities preceded the introduction of these vaccines. The official launch of MR vaccine (including MR2) was presided by the Head of State, raising the visibility of the event, and immensely helping community mobilisation. However, at health facility level, there was some shortage of communication tools.

The cold chain space at national and regional level had been expanded to accommodate the new vaccines. However, at health facility level, 63% of the facilities visited faced shortage of functional cold chain space.

Some of the health facility respondents stated that they started using MCV2 right after the launch while there was some delay in others. The data collection tools including MCV2 were used only after August 2014. More than 40% of the health facilities visited did not have the recording tools and training materials.

### Tanzania

MCV2 was introduced in Tanzania in April 2014, and changed to bivalent measles/rubella vaccine (MR2) after the MR catch-up campaign in October 2014. Planning for the campaign included development and dissemination of Information, Education and Communication (IEC) materials and data recording and reporting tools that were updated to include MR2. Training of health workers was conducted at all levels. Cold chain capacity was assessed and found to be sufficient to handle additional MR doses, except in Zanzibar. The introduction of MR2 did not cause an increase in the frequency of vaccine distribution at any level.

However, only 38% of the health facilities visited during the PIE were offering daily vaccination with measles containing vaccines. Health workers often wait for a high number of children to be present during immunization sessions before opening a vial of MR. Parents/ caretakers and some health workers were not adequately aware of the need for MR2 at 18 months of age.

Monitoring charts tracking MR2 coverage were present in 69% of health facilities surveyed. However, vaccination coverage monitoring charts for MR2 was not uniformly utilized in all health facilities. Not all health workers knew the target population for MR2, and most had challenges with the calculation of drop-out rates. Some health facilities were still using the old version of vaccination dose monitoring and recording tools.

### Zambia

The national immunisation program developed a manual to guide the introduction and updated record keeping tools (including stock cards, children’s cards, registers, tally sheets and databases). Health workers were trained, and cold chain capacity was increased in preparation for MCV2 introduction. However, social mobilization and demand creation activities for MCV2 were limited.

Field visits conducted as part of the evaluation revealed that vaccination data is not systematically recorded in registers, and 50% of health facilities visited did not have vaccination coverage monitoring charts. MCV2 coverage calculation is not consistently understood, drop-out is not regularly monitored and tracking of children receiving MCV1 to ensure that they return for MCV2 is not systematically implemented. In a few health facilities, MCV2 doses were tallied as MCV1.

### Zimbabwe

Zimbabwe introduced MCV2 as MR2 following the nationwide MR catch-up SIAs. Cascaded training was conducted before introduction of the new vaccine. Training of trainers (TOT) was done at the national level, and cascaded down to the provincial and district levels. At least two nurses were trained per health facility before the introduction of the MR vaccine.

Guidelines were developed and distributed to all levels. However, these guidelines were not present in most district health offices and health facilities during the field visit. Some data collection tools (e.g. tally sheet, monthly report forms) are already updated to include MR and MCV2.

The updated data collection tools were not distributed to all districts and health facilities, so some health facilities were still using the old tally sheets and reporting forms. The EPI register and child health card had yet to be updated. Nearly all health facilities visited were using immunization monitoring charts which include the new vaccines.

## Discussion

While MCV1 coverage (weighted average) in the Region had been maintained above 70% from 2009 to 2015, MCV2 coverage has remained below 20% as of 2015. Due to the MCV2 introduction criteria previously in place, only about half of the countries in the Region were able to introduce MCV2, and these countries have had MCV1 coverage levels of more than 80% for several years. However, Niger introduced MCV2 without having met the MCV1 coverage criteria. The fact that these countries had relatively strong EPI programs with high MCV1 coverage was not necessarily reflected in high MCV2 coverage. Fifteen of the 23 countries had MCV2 coverage below 80% and 18 countries had MCV1-MCV2 drop-out rates of more than 10%. Algeria, Cape Verde, Seychelles and Swaziland have MCV2 coverage levels equal to or higher than MCV1 coverage.

Since their beginning in the 1970s, childhood vaccination programs in the African region have focused on a target population of children below 12 months of age. In this context, MCV2 has been introduced as the first childhood vaccine that is being delivered beyond the traditional target age for vaccination. This expands both the size of the cohort and the age of children requiring additional vaccination services. With this expansion, the vaccination program must successfully plan and implement a number of activities including creating awareness and community demand for vaccination services, identifying and tracking children receiving MCV1 to ensure that they also receive MCV2, and verifying vaccination status at each contact with the health system to ensure high coverage.

MCV2 introductions in these countries were not meticulously and rigorously implemented as the findings of the PIEs demonstrated. Furthermore, formal PIEs were not conducted in half of the countries indicating possible gaps in identifying and implementing the corrective action needed to improve MCV2 coverage. The findings from the PIE exercises indicate that, while cold chain and logistics systems were mostly updated to accommodate MCV2, health worker training was of insufficient quality, awareness raising about MCV2 did not translate into increased demand by parents and vaccination coverage monitoring and data collection tools were not systematically updated and integrated effectively in all health facilities. This gap in documenting doses may partly be responsible for the low MCV2 coverage in some of these countries. The hesitancy of health care workers to open vials of MCV vaccine to reduce wastage likely contributes to lower MCV2 as this effectively results in less vaccination opportunities.

Similarly, countries in South America documented various challenges in the process of introducing new vaccines, including insufficient cold chain capacity, limited training, limited communication and social mobilization activities^19^. Zambia has documented challenges related to the adequacy of human resources as well as in the training needs and work load of health workers in its introduction of new vaccines^20^. On the other hand, Rwanda used focus groups, micro-planning workshops, detailed cold chain analyses and planning at least a year in advance, to avoid problems during the rollout of multiple vaccines and to prevent stock-outs^21^.

Adding MCV2 during the second year of life is also an opportunity to increase the coverage for MCV1. Vaccination services in the second year of life will provide more opportunities for health workers to screen and ensure that MCV1 doses have been provided to all eligible children, thus removing the artificial barrier of services provided to only under-1 year old children.

Achieving sustained coverage of ≥95% for both MCV1 and MCV2 is necessary to maintain measles elimination and can reduce the need to conduct periodic mass vaccination campaigns for measles. The WHO guideline for the introduction of new vaccines including MCV2 recommends using this opportunity to retrain the workforce, improve the functional cold chain and storage facilities, as well as develop and implement innovative and effective social mobilization plans that will educate parents on the importance of MCV2 in protecting children from measles infection.

The introduction of an opportunity for a scheduled vaccination visit in the second year of life requires efforts to raise parental awareness and generate demand for vaccination services to drive improved coverage. In addition, health workers should be adequately trained including the correct use of the revised vaccination monitoring and data collection tools. Routine health care visits, as well as the implementation of the integrated management of newborn and childhood illnesses (IMNCI) should be optimally utilized as opportunities to verify a child’s entire routine vaccination status.

With the recent revision of WHO recommendations for MCV2 introduction, the remaining countries in the Region are expected to take steps to introduce MCV2 into their routine immunization programmes in the coming months. Some of these countries have large target populations and annual birth cohorts, and have routine immunization systems that do not achieve high coverage. These countries have had relatively weaker immunisation programs and will be faced with more of the systemic challenges that are described in the countries reviewed in this paper – challenges related to demand creation, immunisation monitoring systems, health worker training, vaccine logistics, drop-out monitoring and service provision, among others. National immunisation programs have to anticipate, and address these challenges in order to take full advantage of the second year of life platform for immunisation.

To ensure a successful MCV2 introduction capable of achieving high coverage, pre-introduction district visits should be undertaken to assure readiness and resolve major bottlenecks that could adversely impact introduction^8^. Countries should ensure that PIE exercises are conducted not long after vaccine introduction in order to implement corrective actions, monitor vaccine wastage rates and MCV1- MCV2 drop-out rates for remedial action, as well as analyze subnational levels of coverage. The WHO African Region should adopt a coverage target for MCV2 as a routine vaccination system performance indicator.

## Limitations

Some of the countries introduced MCV2 following MR SIAs, and others conducted multiple vaccine introductions within the same time period. Moreover, some of these evaluations were done as part of broader EPI program review exercises, and do not necessarily delve deep into specific issues related to MCV2 introduction. This diversity in the approach used for the MCV2 introduction and the evaluations makes it difficult to compare the experiences of countries.

## Figures and Tables

**Table 1:** MCV1 and MCV2 coverage (weighted Regional average). African Region. 2006 – 2015. WHO-UNICEF coverage estimates.

	MCV1	MCV2	Number of countries reporting MCV2
**2006**	64%	5%	6
**2007**	66%	4%	6
**2008**	68%	4%	6
**2009**	73%	5%	6
**2010**	73%	5%	6
**2011**	72%	5%	7
**2012**	72%	6%	9
**2013**	71%	7%	11
**2014**	72%	11%	17
**2015**	74%	18%	23

**Table 2:** MCV1 and MCV2 coverage and drop out rates. WHO-UNICEF coverage estimates. 2015

Country	MCV1	MCV2	MCV1 - MCV2 drop-out rate^1^	Type of vaccine used as of Dec 2016	Age of MCV2 administration in months	# of years since country started reporting MCV2 coverage
**Algeria**	95%	99%	-4.2%	M	6 years	16
**Angola**	55%	26%	52.7%	M	15 months	1
**Botswana**	97%	85%	12.4%	MR	18 months	3
**Burkina Faso**	88%	50%	43.2%	MR	15 months	2
**Burundi**	93%	65%	30.1%	M	18 months	3
**Cabo Verde**	92%	95%	-3.3%	MMR	15 months	5
**Eritrea**	85%	75%	11.8%	M	18 months	1
**Gambia**	97%	77%	20.6%	M	18 months	4
**Ghana**	89%	63%	29.2%	MR	18 months	4
**Kenya**	75%	28%	62.7%	MR	18 months	1
**Lesotho**	90%	82%	8.9%	M	18 months	15
**Malawi**	87%	8%	90.8%	M	15 months	1
**Mauritius**	99%	85%	14.1%	MMR	5 years	12
**Niger**	73%	16%	78.1%	M	16 months	2
**Rwanda**	97%	87%	10.3%	MR	15 months	1
**Sao Tome and Principe**	93%	76%	18.3%	MR	18 months	2
**Senegal**	80%	54%	32.5%	MR	15 months	2
**Seychelles**	98%	98%	0.0%	MMR	6 years	15
**Sierra Leone**	76%	60%	21.1%	M	15 months	1
**South Africa**	76%	63%	17.1%	M	12 months	16
**Swaziland**	78%	89%	-14.1%	MR	15 months	14
**Tanzania**	99%	57%	42.4%	MR	18 months	2
**Zambia**	90%	47%	47.8%	M	18 months	2

1 MCV1 – MCV2 drop-out rate = (MCV1 – MCV2)/ MCV1 coverage

**Table 3:** Timing of post-introduction evaluation in countries introducing MCV2 from 2012 - 2015.

Country	Year of MCV2 introduction	Date of PIE	Interval in Months
**Eritrea**	July-12	Feb-15	31
**Gambia**	Aug-12	September-15	37
**Ghana**	Feb-12	Aug-13	18
**Burundi**	January-13	July-14	16
**Zambia**	July-13	July-14	12
**Kenya**	July-13	May-15	22
**Sao Tome and Principe**	Dec-14	November-13	11
**Senegal**	Aug-14	July-15	12
**Tanzania**	October-14	July-15	9
**Malawi**	July-15	October-16	15
**Zimbabwe**	October-15	July-16	9
